# Clinical Evaluation of the Efficacy, Safety, and In-Use Tolerability of a Diacnemide™-Containing Acne Kit (Facial Serum and Cleanser) Regimen for the Synergistic Management of Facial Acne in Adults

**DOI:** 10.7759/cureus.69968

**Published:** 2024-09-23

**Authors:** Maheshvari N Patel, Nayan K Patel, Apeksha M Merja, Saurav Patnaik

**Affiliations:** 1 Clinical Research, NovoBliss Research Private Limited, Ahmedabad, IND; 2 Pharmacology, Swaminarayan University, Ahmedabad, IND; 3 Dermatology, NovoBliss Research Private Limited, Ahmedabad, IND; 4 Cosmetology, Anveya Living Private Limited, Gurgaon, IND

**Keywords:** acne, dark spots, facial cleanser and serum, iga scoring, niacinamide, porphyrins

## Abstract

Introduction

Acne is a common inflammatory condition characterized by comedones, papules, and pustules, often resulting from increased sebum production influenced by hormones such as insulin-like growth factor-1 and androgens. Factors like *Cutibacterium acnes*, medications, sun exposure, cosmetics, and genetics exacerbate acne. This study aims to assess the safety and effectiveness of a novel acne treatment regimen, including a cleanser and serum containing Diacnemide™ (manufactured by Beaucience India Private Limited, Faridabad, India), in improving acne symptoms in adults.

Methods

This prospective, interventional, open-label, single-center, single-arm clinical study was designed to evaluate the safety, efficacy, and in-use tolerability of the test treatment regimen (serum + cleanser) for facial acne. Ethical approval was obtained from the Independent Ethics Committee, and all participants provided written consent. The study assessed changes in the severity of acne by dermatological assessment using the Investigator's Global Assessment (IGA) scale, changes in inflammatory and non-inflammatory lesions, skin hydration, sebum levels, and facial blemishes using various bioinstrumentations from Courage+Khazaka Electronic GmbH, Germany - Visiopor® PP34N, Corneometer® CM 825, Sebumeter® SM 815, and Mexameter® MX 18, respectively. Evaluations were conducted at baseline, T15 minutes, day 8, and day 15 post-usage. Statistical analyses were performed using SPSS (version 29.0.1.0) and Microsoft® Excel 2019 software, with results reported using p-values and confidence intervals at a 5% significance level.

Results

Porphyrin measurements showed significant reductions over time, with a 27.18% decrease in quantity and a 39.86% reduction by day 15 (p < 0.0001). Porphyrin values dropped by 2.61% after 15 minutes and 7.82% by day 15 (p < 0.05). Skin hydration increased significantly, with a 97.54% increment after 15 minutes and a 102.74% increment by day 15 (p < 0.0001). Sebum levels were restored to normal levels with the dry skin observed at baseline, showing a 34.45% improvement on day 8 and 75.75% by day 15 (p < 0.0001). Facial dark spots were reduced by 10.66% by day 8 and 14.64% by day 15 (p < 0.0001), and erythema levels decreased significantly. Acne severity showed notable improvement, with 50% of the subjects having moderate acne at baseline, reduced to 20.69% with mild acne, and 79.31% with almost clear skin by day 15. Subject responses indicated high satisfaction, with 100% agreeing on the test treatment's effectiveness in reducing acne, oiliness, and inflammation and improving hydration and skin texture.

Conclusion

The ThriveCo acne regimen (serum + cleanser) manufactured by Anveya Living Private Limited, India, is both effective and safe for treating facial acne in healthy adults with acne. It significantly reduces porphyrin size and quantity, indicating a strong inhibition of *P. acnes*, and visibly improves dark spots and skin hydration. The components - Diacnemide™ and zinc pyrrolidone carboxylic acid - in the cleanser regulate sebum production and provide anti-inflammatory benefits, while the serum, containing Diacnemide™ and niacinamide, enhances skin barrier function and balances lipids. The synergistic effects of the ThriveCo Goodbye Acne Cleanser and Serum in this regimen effectively target surface bacteria like *C. acnes* and deeper follicular issues, promoting healthier skin and reducing acne-related symptoms.

## Introduction

Acne, a prevalent and chronic inflammatory condition of the pilosebaceous follicles, affects over 85% of teenagers and can persist into adulthood, with females comprising two-thirds of dermatologist consultations for acne [1,2]. From a clinical perspective, acne manifests as open and closed comedones, papules, and pustules and can lead to significant dermal tissue damage and scarring over time. The production of sebum, an oily substance, is influenced by insulin-like growth factor-1 (IGF-1) and androgens. IGF-1 stimulates the adrenal and gonadal glands to synthesize androgens, which in turn stimulate sebum production, contributing to the development and persistence of acne [3].

Acne develops due to increased sensitivity of the sebaceous glands to typical levels of circulating androgens. This sensitivity is worsened by the presence of *Cutibacterium acnes* (formerly known as *Propionibacterium acnes*), a bacterial species that triggers inflammation in the affected areas [4].

Proposed contributing factors to acne include the use of medications like lithium, steroids, and anticonvulsants; excessive sunlight exposure; wearing occlusive items such as shoulder pads, headbands, backpacks, and underwire bras; the use of oil-based cosmetics and facial massage; and endocrine disorders such as polycystic ovarian syndrome and pregnancy. Premenstrual acne flares are often associated with edema of the pilosebaceous duct in approximately 70% of female patients. Genetic factors also play a significant role in influencing the composition of sebum's branched fatty acids, with heritability estimates ranging from 50% to 90%. In addition, repetitive mechanical trauma from scrubbing affected skin with soaps and detergents can exacerbate acne symptoms [5]. Several studies have suggested that higher consumption of milk and diets with a high glycaemic load are linked to acne during adolescence [6]. Patients with acne commonly present with symptoms such as comedones (blackheads and whiteheads), papules (small red bumps), and pustules (pus-filled lesions) [7].

Acne vulgaris pathogenesis involves androgen-stimulated sebaceous gland activity, dysbiosis of the follicle microbiome, and cellular immune responses, influenced by genetics and diet. It begins with microcomedone formation, evolving into closed comedones (whiteheads), open comedones (blackheads), and inflammatory lesions (papules, pustules, and nodules). Key factors include increased sebum production (seborrhoea), follicular hyperkeratinisation, *C. acnes*, and inflammation. Microcomedones develop into closed comedones with keratin and sebum build-up; open comedones form as the follicular opening enlarges and oxidizes. Inflammatory lesions arise from bacterial proliferation and immune responses, leading to follicular rupture and exacerbated inflammation [8,9].

Common acne treatments typically include azelaic acid, benzoyl peroxide, and salicylic acid. However, the test acne kit offers a novel approach by combining these ingredients into a single product. The kit features Diacnemide™, which contains aqua, propanediol, triethanolamine, azelaic acid, *Hamamelis virginiana* leaf extract, and sodium hyaluronate. In addition, the acne cleanser includes zinc pyrrolidone carboxylic acid, while the acne serum contains niacinamide.

Azelaic acid is a widely recognized topical treatment for acne and is considered a potential first-line therapy for both non-inflammatory and inflammatory forms of the condition. It exerts a triple action against acne, including a keratolytic effect, antimicrobial properties, and anti-inflammatory effects [10,11]. 

Witch hazel (*Hamamelis virginiana* L.), a deciduous tree native to the Atlantic coast of North America, is commonly incorporated into skincare products despite limited scientific evidence supporting its effectiveness in treating acne. Historically, witch hazel has been extensively used for medicinal purposes, with Indigenous Native American communities utilizing its leaves and bark to treat various conditions including fevers, colds, and skin inflammations [12]. The European Medicines Agency (EMA) reported on the consolidated use of hamamelis hydroalcoholic leaf or bark extracts (ethanol 30-60%) for circulatory and skin disorders [13,14].

Nicotinamide, also called niacinamide, offers strong anti-inflammatory effects without the drawbacks of bacterial resistance and systemic side effects. This makes it a promising treatment option for acne vulgaris. Nicotinamide, a type of vitamin B3, is essential and water-soluble, found in various foods [15].

This study aimed to assess the safety, effectiveness, and tolerability of the test acne kit, comprising an acne cleanser and an acne serum in healthy adult individuals experiencing facial acne. Conducted as an open-label, single arm, single-center, prospective, interventional clinical trial, the research sought to investigate changes in parameters such as inflammatory and non-inflammatory lesions, skin hydration, sebum level, facial dark spot, acne severity, and facial photographs.

## Materials and methods

Ethical conduct of the study

This study was conducted in accordance with the principles outlined in the New Drugs and Clinical Trials Rules 2019, ICH guidance E6 (R2) on "Good Clinical Practice," Indian Council of Medical Research (ICMR)'s National Ethical Guidelines for Biomedical and Health Research Involving Human Participants 2017, and the Declaration of Helsinki (Brazil, October 2013). Ethical approval for the study plan was obtained from the ACEAS - Independent Ethics Committee (approval no. NB230034-AL) prior to the commencement of any study-related activities. All participants provided signed informed consent before enrolment in the study. The consent process included a detailed explanation of the study objectives, procedures, confidentiality measures, and the voluntary nature of participation.

Furthermore, this clinical study was registered with the Clinical Trial Registry of India (CTRI) (CTRI/2023/11/060289). This comprehensive ethical framework ensures the study's compliance with international and national ethical standards, safeguarding the rights, safety, and well-being of all participating subjects.

Study design

This was a prospective, interventional, open-label, single-center, single-arm clinical study to evaluate the safety, efficacy, and in-use tolerability of the test treatment. The study was conducted at NovoBliss Research Private Limited, Ahmedabad, India, involving a total of 32 adult subjects aged 18 to 50 years with mild to moderate facial acne. The study duration was 15 days and was funded by the manufacturer (Anveya Living Private Limited).

The primary objective was to evaluate the effectiveness of the test treatment in terms of changes in inflammatory and non-inflammatory lesions (porphyrin size, quantity, and values), skin hydration, sebum level, and facial dark spots (blemishes). The secondary objective was to evaluate the effectiveness of the test product in terms of changes in acne severity, facial photographs, treatment perception of the test treatment, and the occurrence of any adverse events monitored through dermatological evaluation.

Subjects were screened based on predefined inclusion and exclusion criteria to ensure a homogeneous study population. Inclusion criteria included healthy males and non-pregnant/non-lactating healthy females aged 18 to 50 years with mild to moderate acne according to the Investigator's Global Assessment (IGA) score at screening. Participants included females of childbearing potential who had a negative urine pregnancy test and demonstrated good general health based on recent medical history. If participants were using hormonal contraception, they had to have been on it for at least six months prior to the study and agreed to continue this method throughout the study. In addition, all participants were willing and able to follow study protocols, attend specified visits, and provide written informed consent to participate.

Exclusion criteria included subjects with known allergies or sensitivities to the treatment ingredients, a history of alcohol or drug addiction, or the use of other acne control products during the study. Those with skin irritation, open wounds, cuts, abrasions, or any dermatological conditions on the face that could interfere with assessments were excluded. Participants who wore facial makeup on the day of the study visit, had a medication history affecting skin response, or had concurrent skin diseases were also excluded. Additional exclusions were individuals who had taken systemic corticosteroids, antibacterial, or immunosuppressant drugs, or undergone abrasive facial procedures in the past 30 days, those with excessive sun exposure, pregnant or breastfeeding women, or those planning to become pregnant during the study. Also excluded were individuals with medical conditions or medications that, in the Investigator’s judgment, posed an undue risk, those who had used topical steroids, retinoids, or other topical drugs within two weeks prior to study entry, and participants involved in other clinical studies simultaneously.

Study procedure

The study was conducted over three visits with a total treatment duration of 15 days. During visit 1 (day 1), the participants underwent screening, enrolment, and an initial evaluation of the test treatment. Visit 2 (day 8) encompassed the evaluation phase and the test treatment usage period. Finally, visit 3 (day 15) involved final evaluations and marked the end of the study. Details about the test regimen for acne management are provided in Table 1.

**Table 1:** Details about the test regimen and instruments

Name	ThriveCo Goodbye Acne Cleanser	ThriveCo Goodbye Acne Serum	
Mode of Usage	Dispense a coin-sized amount into your palm. Gently massage onto the face using circular motions for one to two minutes. Rinse thoroughly with water.	Begin with a freshly cleansed face. Apply 2-3 drops of the test acne serum to your face and neck, massaging gently until fully absorbed.	
Ingredients	Diacnemide^TM^ (aqua, propanediol, triethanolamine, azelaic acid, *Hamamelis virginiana* leaf extract, sodium hyaluronate), zinc pyrrolidone carboxylic acid	Diacnemide^TM^ (aqua, propanediol, triethanolamine, azelaic acid, *Hamamelis virginiana* leaf extract, sodium hyaluronate), niacinamide	
Frequency	Twice a day (morning and evening)	Twice a day (morning and evening)	
Route of administration	Topical	Topical	
Manufacturer Details			
Test Regimen	ThriveCo Goodbye Acne Cleanser	Anveya Living Private Limited, Bangalore, India	
	ThriveCo Goodbye Acne Serum		
Ingredients	Diacnemide^TM^	Beaucience India Private Limited, Faridabad, India	
	Zinc pyrrolidone carboxylic acid	Solabia Group, Cedex, France	
		Ajinomoto Co., Inc., Tokyo, Japan	
		Salicylates and Chemicals, Mumbai, India	
		COSROMA, Shanghai, China	
	Niacinamide		
		Jubilant Ingrevia, Pennsylvania, USA	
		Lonza Group AG, Basel, Switzerland	
Instruments	Visiopor® PP34N	Courage+Khazaka Electronic GmbH, Cologne, Germany	
	Corneometer® CM 825		
	Sebumeter® SM 815		
	Mexameter® MX 18		

The IGA scale was used to assess the severity of acne. Evaluations were conducted prior to and following treatment with T15 on day 1, day 8, and day 15. The scale categorizes acne severity as follows: Grade 0 represents clear skin without any inflammatory or non-inflammatory lesions; Grade 1 denotes nearly clear skin with rare non-inflammatory lesions and no more than one small inflammatory lesion; Grade 2 indicates mild severity, with some non-inflammatory lesions and a few papules/pustules but no nodular lesions; Grade 3 signifies moderate severity, with numerous non-inflammatory lesions and possibly some inflammatory lesions but no more than one small nodular lesion; and Grade 4 indicates severe acne, with many non-inflammatory lesions and a few inflammatory lesions, possibly including several nodular lesions.

The Visiopor® PP34N camera (from Courage+Khazaka Electronic GmbH, Germany) utilizes specific UV light to visualize fluorescent acne lesions within a designated area of 4 x 5.5 mm. Skin inflammatory and non-inflammatory lesions were assessed using the Visiopor® PP34N on the subject's skin. Porphyrins appear in red, while other detected fluorescents (falling within the greenish/yellowish range) are marked in green. Results are reported in terms of the area covered by these fluorescents as a percentage, along with their count and the mean color intensity (measured on a scale from 0 to 255 in the HSV (hue, saturation, value) color space). Evaluations of the treatment's efficacy were conducted on day 1, both before and 15 minutes after treatment initiation, and subsequently on day 8 and day 15.

Hydration levels were assessed using capacitance measurements of the stratum corneum, the outermost layer of the skin, which serves as a dielectric medium. Changes in skin hydration were reflected in alterations of its dielectric properties. The Corneometer® CM 825 gauges (from Courage+Khazaka electronic GmbH, Germany) these changes by detecting variations in the dielectric constant, thereby altering the capacitance of a precise capacitor. The evaluation focused on the right cheek of the subjects, with measurements taken on day 1 before and 15 minutes after initiating the test treatment, as well as on day 8 and day 15, using the Corneometer® CM 825.

The Sebumeter® SM 815 (from Courage+Khazaka Electronic GmbH, Germany) is a globally recognized instrument for measuring sebum (oil) levels on the skin, scalp, and hair. It utilizes grease spot photometry, where an opaque tape is applied to the skin or hair surface. The tape's transparency changes based on the amount of sebum present in the measured area. Inserting the tape into the device's aperture allows a photocell to measure this transparency, indicating the sebum content via light transmission. Skin sebum levels were assessed on the subject's right cheek. Evaluations were conducted on day 1 before and 15 minutes after starting the test treatment, as well as on day 8 and day 15, using the Sebumeter® SM 815.

**Figure 1:**
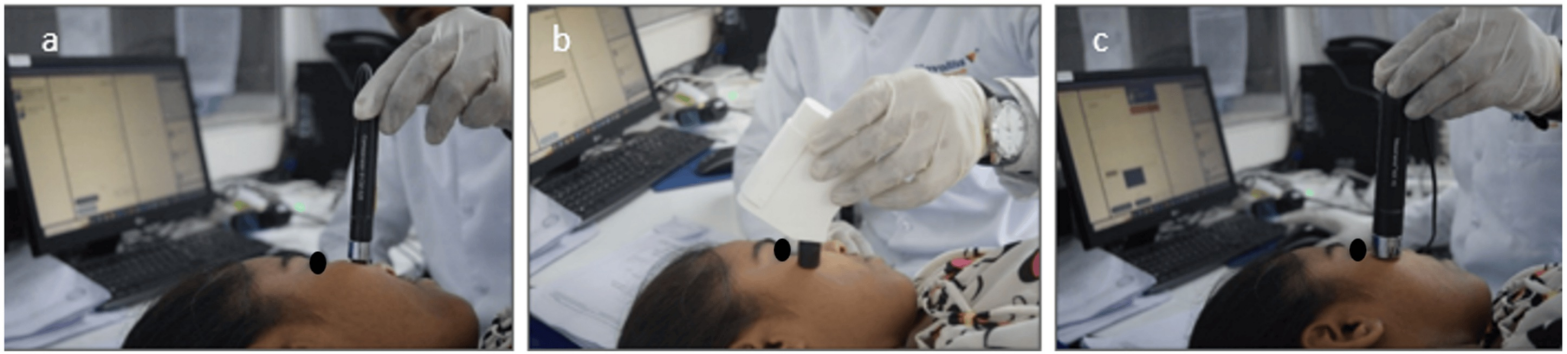
(a) Corneometer® CM 825, (b) Sebumeter® SM 815, (c) Mexameter® MX 18

The Mexameter® MX 18 probe (from Courage+Khazaka Electronic GmbH, Germany) emits three specific wavelengths of light, and a receiver detects the reflected light from the skin. The measurement relied on absorption and reflection principles. By knowing the amount of light emitted, it becomes possible to calculate how much light is absorbed by the skin. Facial blemishes, such as dark spots, were assessed in a chosen area of the subject's face. Evaluations were conducted on day 1 before and 15 minutes after initiating the test treatment, as well as on day 8 and day 15, using the Mexameter MX 18 (Figure 1).

Study-specific digital photographs were taken of the subject’s face (right, left, and front) on day 1 before and after test treatment usage at T15 min, day 8, and day 15 by using a Nikon D3300 digital camera (Nikon Corp., Japan).

Statistical analysis

Continuous variables were described by descriptive statistics (N, mean, SD, median, minimum, and maximum). Categorical variables were expressed by frequency and percentage along with graphical presentations whenever required.

The statistical analysis was done using IBM SPSS Statistics for Windows, Version 29.0.1.0 (released 2012, IBM Corp., Armonk, NY) and Microsoft® Excel 2019 software (Microsoft Corp., USA) with a 5% level of significance. Withdrawn subjects were not included in the statistical analysis.

Data handling and analysis

All data were carefully reviewed and cleaned before analysis to ensure accuracy and completeness. Frequency analyses and cross-tabulations were performed to ensure data accuracy and consistency. Missing data were addressed through appropriate imputation methods or excluded from the analysis, depending on the extent and nature of the missingness. The results of the statistical tests, including p-values, were reported with corresponding confidence intervals to provide a measure of precision and reliability.

Sample size determination

A sample size calculation was performed to ensure sufficient power to detect clinically meaningful differences. Based on this calculation, 32 subjects were enrolled to ensure the completion of 28 subjects.

## Results

Demographic and other baseline characteristics

**Figure 2:**
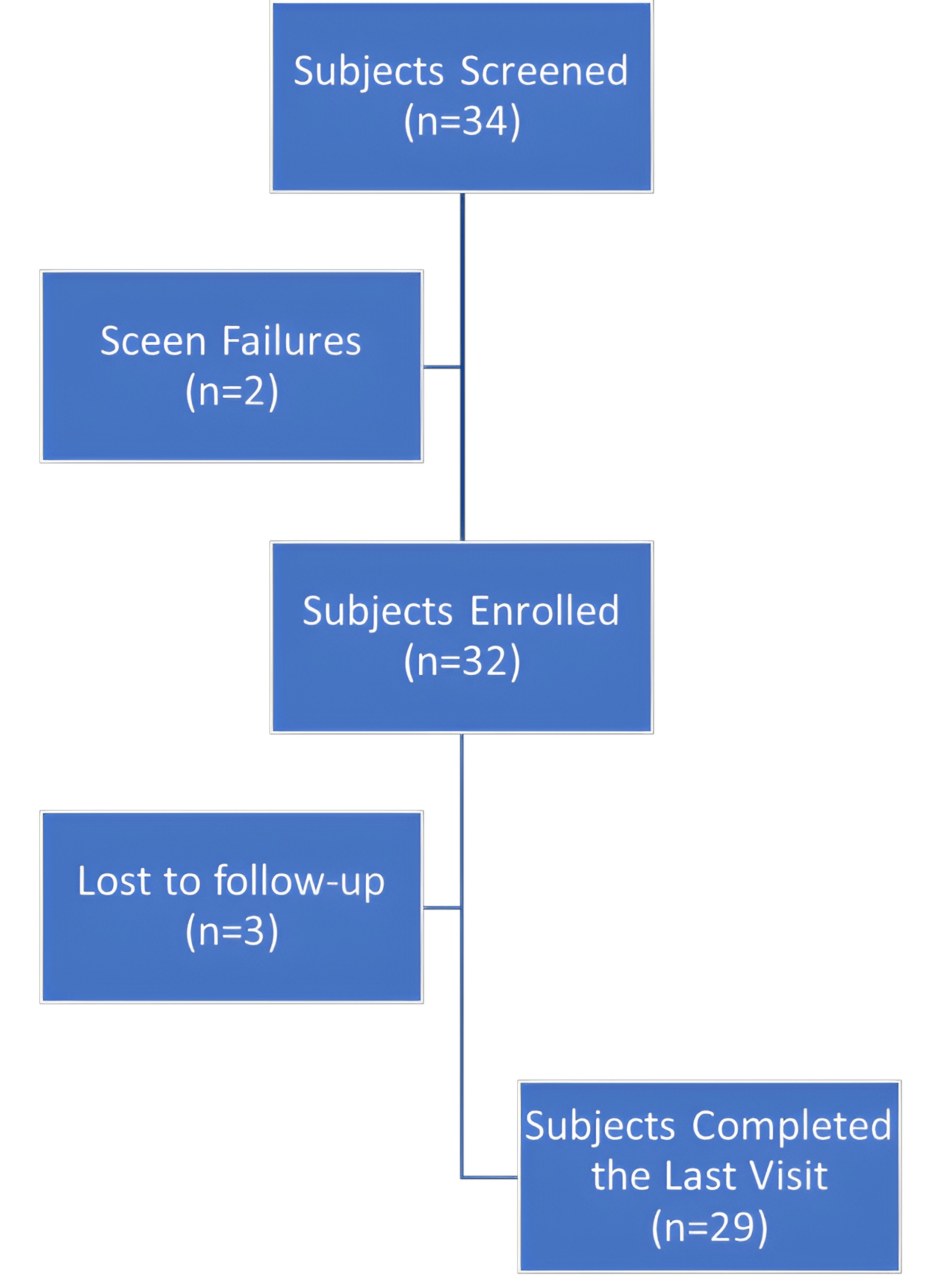
Subject disposition flowchart: overview of the subject enrolment, follow-up, and analysis

A total of 32 subjects, aged 18 to 50 years, were enrolled in the study, with 29 completing it. Despite three dropouts, complete data was collected for both pre- and post-facial acne assessments, ensuring robust analysis. The study maintained high compliance with the intervention and assessment schedules. Refer to Figure 2 for the subject disposition.

The cohort included both female and male subjects, providing a balanced representation, with 53.13% female (n = 17) and 46.88% male (n = 15) participants. The mean age of the participants was 27.16 ± 7.51 years. In addition, the mean height was 157.56 ± 10.83 cm, and the mean weight was 59.53 ± 12.51 kg.

Primary endpoints

Porphyrin Size, Quantity, and Values Evaluated Using Visiopor PP34N

Porphyrin measurements using the Visiopor® PP34N showed significant reductions over time. The initial porphyrin quantity measured 22.79 ± 12.53, decreasing to 16.69 ± 10.80 (27.18% reduction, p < 0.0001) 15 minutes after applying the test product, further reduced to 17.81 ± 10.61 (26.46% reduction, p < 0.0001) by day 8, and to 13.59 ± 9.06 (39.86% reduction, p < 0.0001) by day 15.

**Figure 3:**
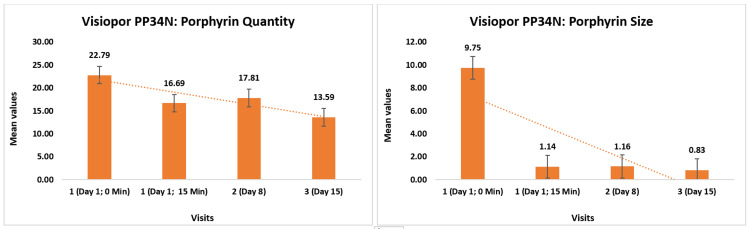
Change in porphyrin quantity and size as assessed by Visiopor® PP34N

In addition, the porphyrin size initially was 9.75 ± 43.91%, reduced to 1.14 ± 1.02% (32.26% reduction, p > 0.05) after 15 minutes, further to 1.16 ± 1.03% (22.81% reduction, p < 0.05) by day 8, and 0.83 ± 0.68% (42.41% reduction, p > 0.05) by day 15 (Figure 3).

**Figure 4:**
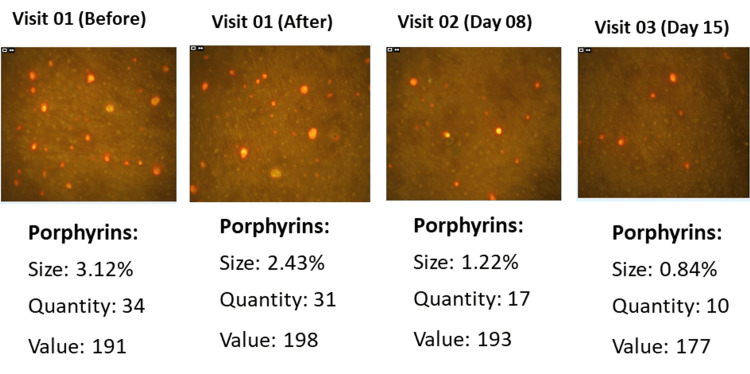
Visiopor® PP34N – reduction in porphyrin levels

The porphyrin value began at 150.17 ± 21.51 and dropped to 145.55 ± 19.48 (2.61% reduction, p < 0.05) after 15 minutes, with a further decrease to 149.42 ± 21.69 (1.11% reduction, p < 0.05) by day 8, and 137.86 ± 20.64 (7.82% reduction, p < 0.05) by day 15 (Figure 4). 

Skin Hydration Using Corneometer® CM 825

Skin hydration, evaluated using the Corneometer® CM 825, showed significant improvements. Initially, it measured 27.68 ± 8.19%, which increased to 51.59 ± 9.74% (97.54% increment, p < 0.0001) after 15 minutes of applying the test product. Subsequent increases from baseline in skin hydration were observed on day 8 (40.30 ± 7.52%, 56.89% increment, p < 0.0001) and day 15 (52.23 ± 8.75%, 102.74% increment, p < 0.0001).

Skin Sebum Level Using Sebumeter® SM 815

Skin sebum levels, measured with the Sebumeter® SM 815, also showed notable changes. Initially, it measured 32.36 ± 16.26 units, increasing to 40.27 ± 17.51 units (34.45% increment, p < 0.0001) on day 8, and further increased to 48.45 ± 18.29 units (75.75% increment, p < 0.0001) by day 15. The sebum levels were observed to normal levels on day 15 from the dry skin observed on day 1 at baseline.

Facial Dark Spots Using Mexameter® MX 18

**Figure 5:**
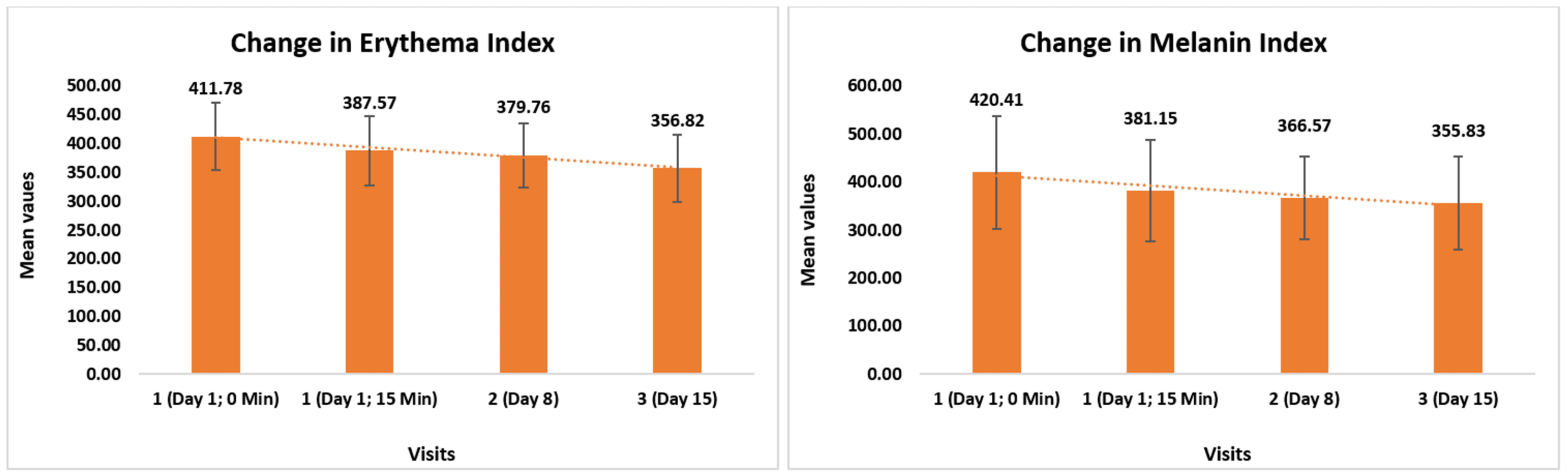
Change in skin erythema and facial dark spots (melanin) assessed by Mexameter® MX 18 The mean values of the Erythema Index and Melanin Index are presented in arbitrary units.

Change in erythema was evaluated using the Mexameter® MX 18. Initially, it measured 411.78 ± 58.16 and decreased to 387.57 ± 60.32 (5.95% reduction, p < 0.0001) after 15 minutes following the application of the test product. There was a further reduction to 379 ± 55.99 (6.89% reduction, p < 0.0001) by day 8 and to 356 ± 58.52 (12.89% reduction, p < 0.0001) by day 15. Change in facial dark spots (melanin) was also evaluated using the Mexameter® MX 18. Initially, it measured 420.41 ± 117.40 and decreased to 381.15 ± 105.24 (8.90% reduction, p < 0.0001) after 15 minutes. Subsequently, there was a further reduction to 366 ± 85.83 (10.66% reduction, p < 0.0001) by day 8 and to 355.83 ± 97.76 (14.64% reduction, p < 0.0001) by day 15 (Figure 5).

Secondary endpoints

Acne Severity Using the IGA Scale

**Figure 6:**
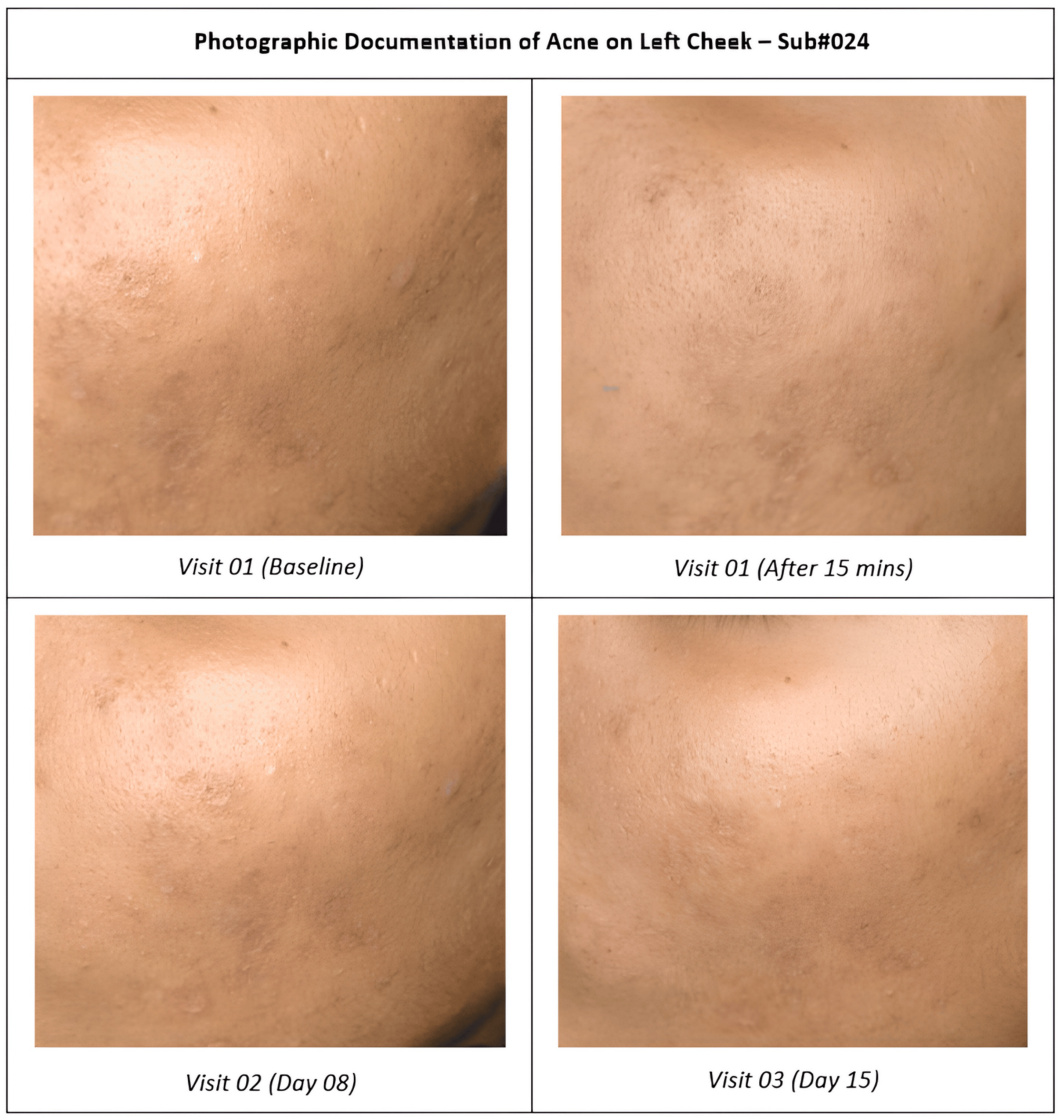
Change in acne severity by the visual dermatological assessment

At baseline, 50% of the subjects had moderate acne and 50% had mild acne. By day 8, the proportion of subjects with moderate acne reduced to 7.69%, while 53.85% had mild acne and 38.46% had almost clear skin. After 15 days of using the test treatment, 20.69% of the subjects had mild acne and 79.31% had almost clear skin. These results demonstrate a clinically significant reduction in acne severity following the use of the test treatment, indicating its efficacy in improving acne conditions. Photographs in Figure 6 show a reduction in acne severity on the visual dermatological assessment.

Change in the Subject Response Index Using the Nine-Point Hedonic Scale

This nine-point hedonic scale includes questions assessing the effectiveness of the test product in areas such as acne improvement, oiliness reduction, inflammation reduction, skin hydration, texture softness and smoothness, and overall satisfaction. Respondents rate each aspect from 1 to 9, where 1 means "extremely dissatisfied," 2 means "very dissatisfied," 3 means "moderately dissatisfied," 4 means "slightly dissatisfied," 5 means "don't know," 6 means "slightly satisfied," 7 means "moderately satisfied," 8 means "very satisfied," and 9 means "extremely satisfied."

Regarding prior product usage, 3.45% of the subjects had used another product previously, while 96.55% had not. For acne improvement, 3.45% neither agreed nor disagreed that the earlier product improved their acne condition, but after 15 days of using the test treatment, 100% agreed it effectively reduced acne. In terms of oiliness reduction, 3.45% of the subjects agreed their earlier product reduced oiliness, whereas 100% agreed that the test treatment reduced oiliness after 15 days. For inflammation reduction, 3.45% agreed the earlier product was effective, while 100% agreed the test treatment reduced acne-related inflammation. Regarding skin hydration, 3.45% felt the earlier product improved skin hydration, but 100% agreed the test treatment enhanced skin hydration. For texture softness and smoothness, 3.45% felt the earlier product provided a soft and smooth texture, compared to 100% who agreed the test treatment did so. Lastly, for overall satisfaction, 3.45% were satisfied with the earlier product, while 100% reported satisfaction with the test treatment after 15 Days.

Safety assessment via dermatological evaluation

The safety of the test product was assessed through dermatological evaluations conducted throughout the study period. The occurrence of any adverse events, such as skin irritation, allergic reactions, or other dermatological issues, was monitored closely to ensure the safety of the participants. The results of the safety assessment revealed that no adverse events were reported during the study. All subjects tolerated the test product well, with no instances of dermatological complications or negative reactions, indicating the safety of the test product for use in managing the targeted skin conditions.

## Discussion

Acne remains a significant dermatological concern worldwide, affecting individuals of all ages and posing challenges to treatment efficacy and patient satisfaction. This study investigated the safety, efficacy, and tolerability of the test acne kit comprising the acne cleanser and acne serum in treating facial acne in healthy adults. Conducted as an open-label, single-arm, single-center clinical trial, the study assessed parameters including inflammatory and non-inflammatory lesions, skin hydration, sebum levels, facial dark spots, acne severity, and facial photographs. The dermatological evaluation in this study was conducted using a validated methodology to ensure accuracy and consistency in clinical readings [15].

Nicotinamide, or niacinamide, is a form of vitamin B3 known for its skincare benefits, particularly in reducing inflammation, which makes it effective for acne, rosacea, and other inflammatory conditions. Niacinamide enhances the skin's barrier function by stimulating ceramide production, thereby reducing water loss and keeping the skin hydrated. In the acne serum, niacinamide plays a crucial role in boosting skin hydration by stabilizing the barrier and maintaining moisture balance, which is vital for acne-prone skin to stay hydrated without increasing oiliness. By reducing water loss and enhancing moisture retention, niacinamide helps maintain optimal skin hydration levels, which is particularly important in acne-prone skin where hydration without exacerbating oiliness is crucial for overall skin health [16,17].

In 2022, Piazza S et al. investigated the biological effects of witch hazel (*Hamamelis virginiana* L.) bark glycolic extract against inflammation induced by *C. acnes*. Their phytochemical analysis identified hamamelitannin and oligomeric proanthocyanidins as the primary constituents. The extract demonstrated inhibition of *C. acnes*-induced IL-6 release and partially suppressed NF-κB activation, despite lacking antibacterial and antibiofilm properties. The extract exhibited stronger anti-inflammatory effects by reducing IL-8 release compared to hamamelitannin, likely due to its high content of proanthocyanidins and antioxidant activity. In our study, we observed that the application of this extract for 15 days resulted in reduced pore size and smoother skin, alleviating acne-related inflammation [18].

A study by Chilicka K et al. compared azelaic and pyruvic acid peels for treating acne in young women aged 18-25 with mild to moderate acne. Both treatments significantly reduced acne symptoms over six sessions, as measured by the Hellegren-Vincent Severity Symptoms scale. In our current study, we evaluated a new treatment's effectiveness in reducing acne severity. Starting with 50% of participants having moderate acne, the treatment reduced this to 7.69% by day 8 and further improved to 20.69% with mild acne and 79.31% nearly clear skin by day 15. These findings demonstrate the treatment's clinical efficacy in improving acne severity according to the IGA scale [19].

The study has several limitations that should be considered. First, the small sample size and the short duration of 15 days may limit the generalizability and long-term applicability of the findings. Furthermore, while various metrics were assessed, the study did not explore other factors influencing acne, such as diet or stress. As no subjects in this study were on oral contraceptives, hormonal profiling was excluded. However, future studies incorporating hormonal profiling of female participants could provide more conclusive insights into the role of hormonal factors in acne treatment outcomes. Despite these limitations, the test acne kit offers a promising treatment option for facial acne in healthy adults. The synergistic effects of the kit's components, particularly the combination of Diacnemide™, zinc pyrrolidone carboxylic acid, and niacinamide, address both the superficial and deeper aspects of acne pathophysiology. This dual-action approach not only reduces acne severity but also contributes to overall skin health by enhancing hydration and reducing pigmentation issues. Future studies could expand on these results by exploring the long-term benefits and potential applications of the test regimen for acne treatment in different populations, providing a more comprehensive understanding of its efficacy and broadening its potential use in clinical practice.

## Conclusions

The ThriveCo Acne Kit has demonstrated both efficacy and safety in treating facial acne in healthy adults. Significant reductions in porphyrin size, quantity, and values suggest that the kit effectively inhibits the growth of *P. acnes*, a primary contributor to acne. In addition, the treatment resulted in visible improvements in dark spots and skin hydration. The kit's components, including Diacnemide™ and zinc pyrrolidone carboxylic acid in the ThriveCo Goodbye Acne Cleanser, regulate sebum production and provide anti-inflammatory benefits crucial for preventing acne and maintaining clear pores. Meanwhile, the ThriveCo Goodbye Acne Serum, containing Diacnemide™ and niacinamide, enhances acne management by improving skin barrier function and balancing lipid levels. This dual approach, utilizing both the cleanser and serum, effectively targets surface bacteria and deeper follicular issues, promoting healthier skin and potentially alleviating acne-related symptoms such as redness and dryness. The synergistic effects of these components offer a comprehensive solution for managing acne and improving overall skin health.
